# Deep learning of wrist accelerometry from UK Biobank data identifies early movement signatures of knee osteoarthritis up to 5 years before diagnosis

**DOI:** 10.1002/ksa.70332

**Published:** 2026-02-17

**Authors:** Ricardo Smits Serena, Michael T. Hirschmann, Georg Matziolis, Rüdiger von Eisenhart‐Rothe, Daniel Rueckert, Florian Hinterwimmer, Christina Valle

**Affiliations:** ^1^ Department of Orthopaedics and Sports Orthopaedics TUM University Hospital Munich Germany; ^2^ Institute for AI and Informatics in Medicine TUM University Hospital Munich Germany; ^3^ Kantonsspital Baselland Bruderholz Switzerland; ^4^ Kantonsspital Baselland Liestal Switzerland; ^5^ Kantonsspital Baselland Laufen Switzerland; ^6^ Waldkliniken Eisenberg GmbH Eisenberg Germany; ^7^ Department of Computing Imperial College London London UK

**Keywords:** accelerometer, knee, machine learning, osteoarthritis, wearable

## Abstract

**Purpose:**

This study aims to test whether week‐long wrist accelerometry combined with deep learning can (i) distinguish healthy individuals from people with knee osteoarthritis (KOA), (ii) separate prodromal KOA from established KOA, and (iii) identify individuals who will receive a KOA diagnosis within 5 years.

**Methods:**

We conducted a retrospective case–control study using the UK Biobank data set. After quality control, 102,120 participants with valid accelerometry were available; KOA was identified via ICD‐10 M17.x codes (*n* = 7262). To reduce adiposity confounding, analyses were restricted to body mass index (BMI) ≥ 29, with controls matched to KOA on age, sex and BMI distributions. We used preprocessed, orientation‐independent, hourly mean acceleration over a 24‐h cycle and included month, sex, age and weight as covariates. A 1D convolutional neural network modelled daily activity profiles with embeddings for categorical covariates. Fivefold cross‐validation assessed accuracy, macro F1, macro sensitivity and AUC.

**Results:**

Balanced cohorts were formed for three tasks: healthy versus KOA (*n* = 3677 per class), prodromal versus diagnosed KOA (*n* = 1596 vs. 2081), and healthy vs prodromal within 5 years (*n* = 1369 per class). Daily activity patterns were similar across groups, with slightly lower daytime acceleration in KOA/prodromal participants. Model performance was moderate for healthy versus KOA (accuracy 63.5 ± 1.2%; AUC 0.672 ± 0.017) and healthy vs prodromal within 5 years (64.5 ± 0.5%; AUC 0.675 ± 0.019). Discrimination between prodromal and diagnosed KOA was close to random (54.6% ± 1.5%; AUC 0.552 ± 0.015).

**Conclusions:**

One week of wrist‐worn accelerometry contains a reproducible signal associated with KOA and can flag elevated risk up to 5 years before diagnosis. Since existing KOA cannot be distinguished from prodromal KOA, it can be assumed that patients show altered movement patterns years before diagnosis. These findings highlight the clinical relevance of early, unobtrusive movement monitoring and support the potential of wearables as a scalable, low‐cost component of population‐level KOA screening.

**Level of Evidence:**

Level II, prognostic study—lower‐quality prospective cohort. The study uses a large, population‐based prospective cohort (UK Biobank) with retrospective analytical methods; follow‐up is high, but the study is a secondary analysis rather than a primary prospectively designed prognostic trial.

**Abbreviations**
AI: artificial intelligence
AUC: area under the curve
BMI: body mass index
CNN: convolutional neural network
ICD‐10: International Classification of Diseases
IMU: inertial measurement unit
KOA: knee osteoarthritis
KOOS: Knee Injury and Osteoarthritis Outcome Score
ML: machine learning
MSD: musculoskeletal disorders
OA: osteoarthritis
ReLU: rectified linear unit
TKA: total knee arthroplasty

## INTRODUCTION

Musculoskeletal disorders (MSDs), especially knee osteoarthritis (KOA), encompass a wide range of conditions affecting muscles, bones and joints, affecting millions of people globally [7]. Their onset and progression can be influenced by various factors, ranging from physical attributes to lifestyle choices [7, 15]. KOA is a demographically driven, increasingly common condition that in its advanced stages is ultimately treated with a total knee arthroplasty (TKA). Therefore, a rising number of primary TKA surgeries and a rapidly growing number of TKA revisions have been projected in the next decades, which will provide a huge challenge for the future healthcare systems [9].

Because KOA progresses slowly, early detection and treatment are preferable [19]. In practice, however, most patients seek care only after they develop marked symptoms such as nighttime pain, persistent pain, or major functional impairment [1]. The period during which KOA develops before the first clinical visit is generally unrecognized, and objective data on movement intensity and quality in the preceding weeks or months are rarely available. Longitudinal monitoring of individuals' movement could reveal subtle changes in daily activity patterns and help identify chronic conditions like KOA before prominent clinical signs emerge. This would allow diagnostic evaluation and therapy to begin earlier, potentially delaying disease onset or progression and preserving patients' long‑term quality of life.

To gather longitudinal movement data, a practical and user‐friendly mobile tool is essential. Accelerometers, alone or combined with additional sensors, are particularly well suited for this purpose [10]. Their data support immediate applications such as biofeedback‐assisted therapy and enable long‐term analysis of large cohorts for cross‐site monitoring or predictive modelling. Because of their compact size, accelerometers can be embedded in wearable devices, making them readily usable in both clinical and research environments [10, 21]. These sensors are present in nearly all smartphones and smartwatches, and they can also be attached to clothing or dedicated bands.

In KOA, accelerometry has been applied across a wide range of clinically relevant use cases, including the characterization of gait features and joint biomechanics, risk assessment, patient stratification, symptom classification and monitoring of functional decline [2, 3, 5, 6, 11, 22, 23, 24]. Moreover, Schalkamp et al. demonstrated that accelerometry outperformed genetic, lifestyle, blood‐biomarker and prodromal‐symptom data in identifying both diagnosed and prodromal Parkinson's disease up to 7 years before clinical onset [20].

Although inertial measurement technologies such as accelerometers are increasingly used in neurology and geriatrics for diagnostics, therapy and prevention, their application in orthopaedics remains relatively underexplored, with only 6.5% of studies being done in orthopaedics [21]. In the field of KOA, there are various studies on the prediction of individual parameters relating to KOA, as mentioned above, but not on the prediction of KOA itself. Leveraging machine learning (ML) with large‐scale accelerometer data sets may offer a promising approach for detecting KOA at its earliest stages.

This study aims to develop and validate a deep learning model capable of distinguishing between healthy individuals, prodromal KOA cases, and patients with established KOA using daily wrist‐worn accelerometer data from the UK Biobank. The objective is to determine whether patterns in everyday movement can serve as early indicators or predictors of KOA onset. We hypothesize that daily wrist‐worn accelerometry contains movement patterns that differ between these groups and may offer measurable signals associated with early KOA development.

## METHODS

### Study design

This study utilized a retrospective, case–control design using accelerometer data from the UK Biobank to identify and predict KOA status and progression. The study was structured around three predictive scenarios: (1) differentiating healthy participants from all KOA patients (both clinically diagnosed KOA and prodromal KOA), (2) distinguishing prodromal KOA from patients already diagnosed with KOA, and (3) predicting future diagnosis within 5 years by comparing healthy participants with individuals in a prodromal stage. ‘Prodromal’ was defined as participants who had not yet been diagnosed at the time of the accelerometry measurement and received a diagnosis within 10 years.

### The UK Biobank data set

A suitable large‑scale data set for developing a high‑performing machine‑learning model is the UK Biobank. The UK Biobank is a biomedical database that contains de‑identified genetic and health information on half a million participants aged 40–69 at recruitment and residing in the United Kingdom  [1, 4]. All UK Biobank participants provided written informed consent for the use of their data in health‑related research, and the UK Biobank has obtained ethical approval from the relevant UK Research Ethics Committee (Application ID 144927). This research has been conducted using the UK Biobank resource under application number 144927. Participants have been followed longitudinally, and the resource includes detailed data on demographics, accelerometer‑derived movement patterns, physical measurements, comorbidities and self‑reported medical conditions.

**Figure 1:**
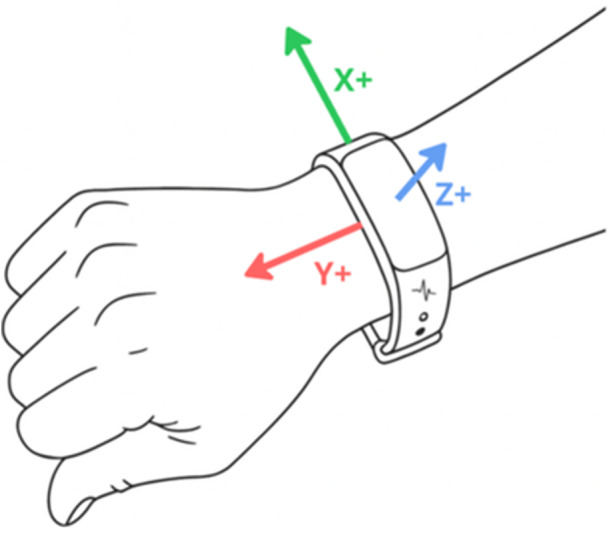
Axis orientation of the axivity AX3 triaxial accelerometer. The diagram illustrates the coordinate system relative to the wrist when the device is worn. The red arrow (Y+) represents the lateral axis, the green arrow (X+) represents the longitudinal axis pointing towards the hand, and the blue arrow (Z+) represents the vertical axis perpendicular to the device face.

These data offer valuable insight into the complex factors that influence MSDs such as KOA. Within the UK Biobank, 103,712 accelerometer recordings were obtained, with a median wear time of 6.9 days, providing valid information for physical‑activity analyses  [4]. Activity was measured using the Axivity AX3 wrist‑worn triaxial accelerometer, a commercial version of the Open Movement AX3 sensor developed by Open Lab at Newcastle University. The device was worn on either the left or right wrist, resulting in differing axis orientations depending on the side of wear (see Figure 1).

### Participants

Participants were drawn from the UK Biobank data set, restricted to individuals with complete accelerometer records and without anomalously high readings exceeding 1000 m/s^2^ (*n* = 102,120). KOA patients were identified through ICD‐10 diagnosis codes M17, M17.0, M17.1 and M17.9, yielding a final cohort of 7262 patients. To minimize potential confounding by adiposity, inclusion in both groups required a body mass index (BMI) ≥ 29. This threshold was chosen because it corresponded to the mean BMI of the KOA cohort, and exploratory analyses indicated that BMI exerted the strongest influence on movement measures up to this value, thereby improving comparability above the cutoff. Controls were matched to KOA patients by gender, age, and BMI distributions to enhance demographic comparability. Exclusion criteria included missing accelerometer data, anomalous accelerometer readings (>1000 m/s^2^) or BMI < 29.

### Data preprocessing

Accelerometer data from the UK Biobank had already undergone standardized preprocessing using established pipelines (e.g., GGIR) [17]. Raw triaxial signals were calibrated, filtered and processed to detect non‐wear, after which validated physical activity and sleep measures were derived [4]. For this study, we used the processed data set provided by the UK Biobank, which summarized accelerometer recordings as average acceleration values in hourly intervals across the 24‐h cycle.

**Figure 2:**
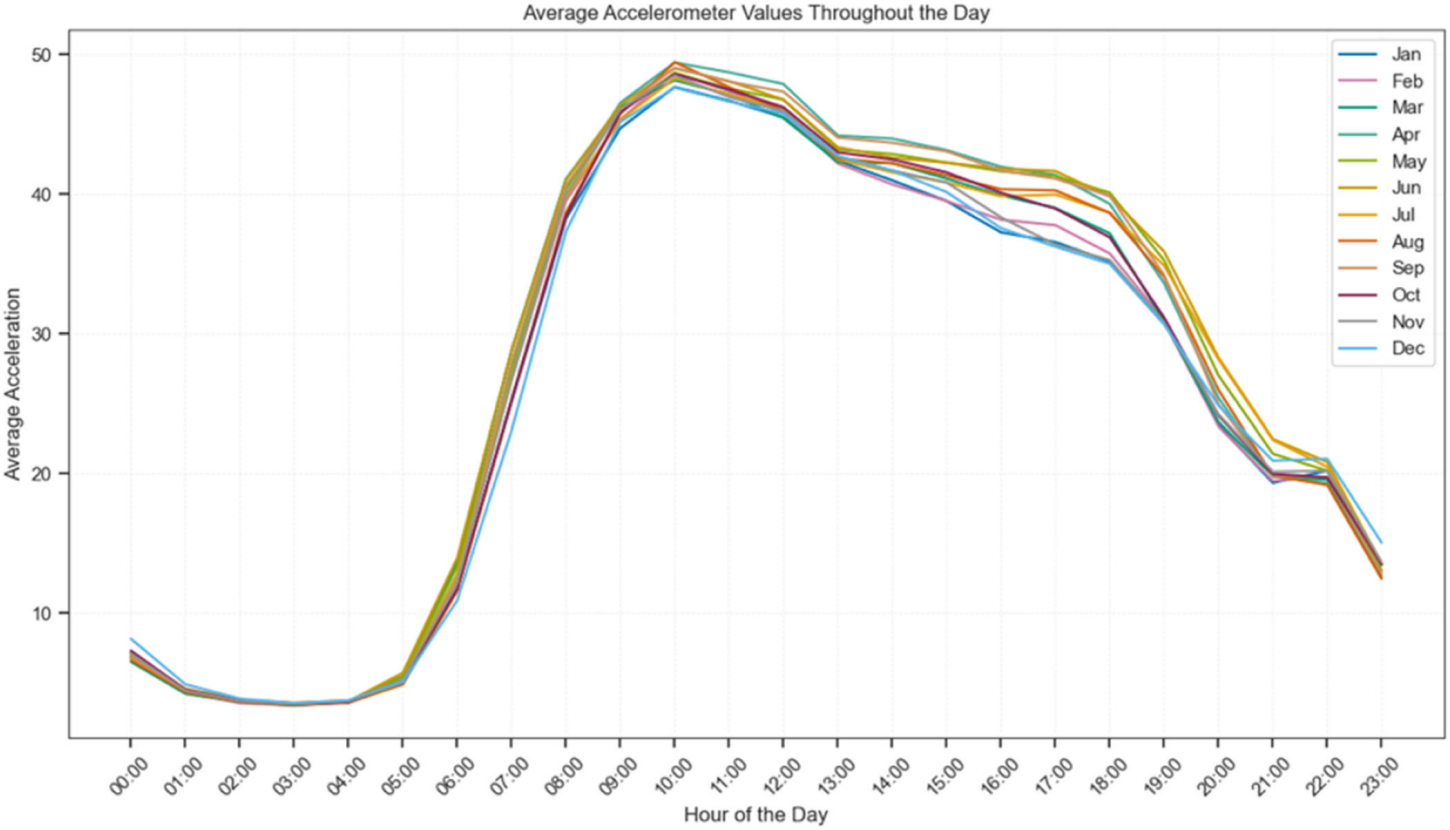
Average accelerometer values throughout the day, stratified by month. The trajectories illustrate seasonal variations in activity, with generally lower average acceleration observed during winter months compared to summer months.

Alongside these activity measures, demographic and contextual variables were extracted, including age at recording, gender, weight and month of recording. Month was specifically included to account for seasonal variation in activity, as lower activity levels were consistently observed in winter compared to summer months across participants (see Figure 2).

For predictive modelling, data were structured into three comparative classes:

1. Healthy versus KOA (prodromal + diagnosed),
2. Prodromal KOA versus already diagnosed KOA, and
3. Healthy versus prodromal KOA cases who subsequently received a KOA diagnosis within 5 years.

Preprocessing involved detailed structuring and standardization of the accelerometer data, removing records with missing values or abnormal measurements. Sample sizes for the comparisons involving healthy controls (Scenarios 1 and 3) were determined by the number of available disease cases to prevent class imbalance. Healthy controls were undersampled to match the specific disease group in a 1:1 ratio based on age, gender, and BMI similarity. Conversely, for the comparison between disease stages (Scenario 2), all eligible participants were retained to maximize the available data. Matching between the KOA and control groups was achieved using weighted sampling to balance age, gender, and BMI distributions, thereby reducing demographic confounding. The final data set therefore consisted of 24‐hourly averaged accelerometer features combined with demographic and contextual variables.

### Predictive modelling

Since the data consisted of short, fixed 24‐point hourly profiles, the problem had an inherently temporal and circadian structure. Traditional models such as random forests handle tabular features well but struggle to capture local daily patterns without custom feature engineering, whereas a compact 1D‐CNN with 24‐aware pooling can target these rhythms while natively fusing contextual inputs, month and gender via embeddings, age and weight via simple projections, within one architecture [8].

**Figure 3:**
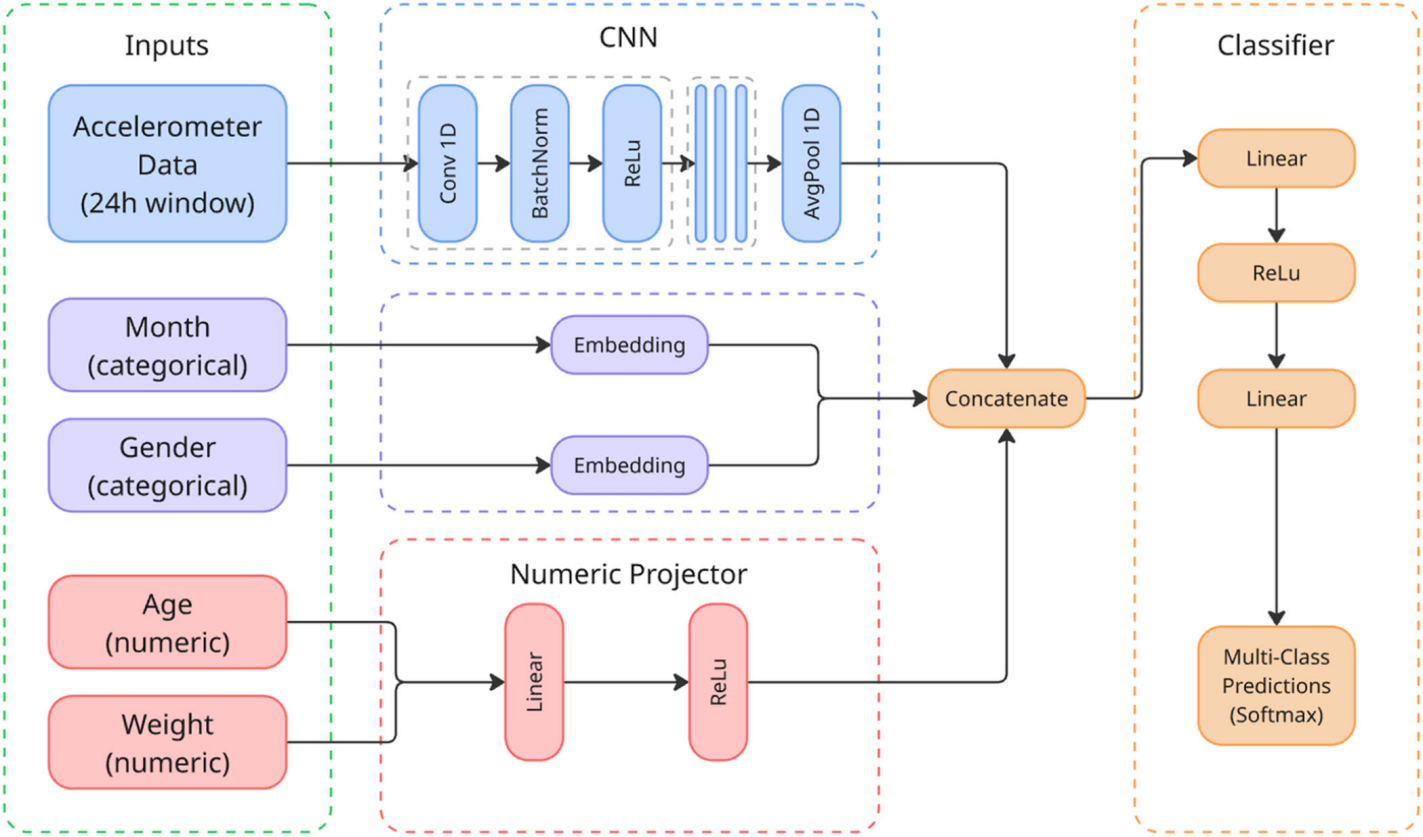
Architecture of the multi‐modal neural network. The model integrates accelerometer data (processed via 1D CNN), categorical variables (via embeddings) and numeric demographics (via a linear projector). These feature representations are concatenated and passed through a final classifier to generate multi‐class predictions. CNN, convolutional neural network.

The CNN consisted of two sequential convolutional layers (16 and 32 filters, respectively), each followed by batch normalization and a rectified linear unit (ReLU). The output was pooled using average pooling over 24‐h sequences. Additional embedding layers were included to process the categorical variables (month of recording and gender). A numeric projector was used to process the demographic features (age and weight) through a fully connected layer with ReLU activation. These distinct feature sets were then concatenated and passed through a final classifier, consisting of two linear layers separated by a ReLU activation, producing the final predictions across multiple classes (see Figure 3). Model training and evaluation were performed using a stratified fivefold cross‐validation scheme to assess generalizability. The network was trained for 30 epochs per fold with a batch size of 64 and a learning rate of 0.001. All analyses were implemented in Python using Scikit‐learn for performance metrics and custom pipelines for feature integration.

### Data analysis

Statistical analyses and predictive modelling were conducted using the Python programming language, specifically libraries such as PyTorch for convolutional neural network (CNN) implementation and scikit‐learn for model evaluation. Model performance was evaluated using accuracy, macro F1‐score, sensitivity, area under the receiver operating characteristic curve (AUC), and confusion matrices. To ascertain the statistical significance of the classification results, a chi‐squared test of independence was performed on the confusion matrix to determine if the association between predicted and true labels was significant. Furthermore, a one‐sample proportion *z* test was used to compare the model's overall accuracy against the 50% baseline expected from random chance in a balanced data set. For all analyses, a *p* value of less than 0.05 was considered statistically significant.

## RESULTS

**Figure 4:**
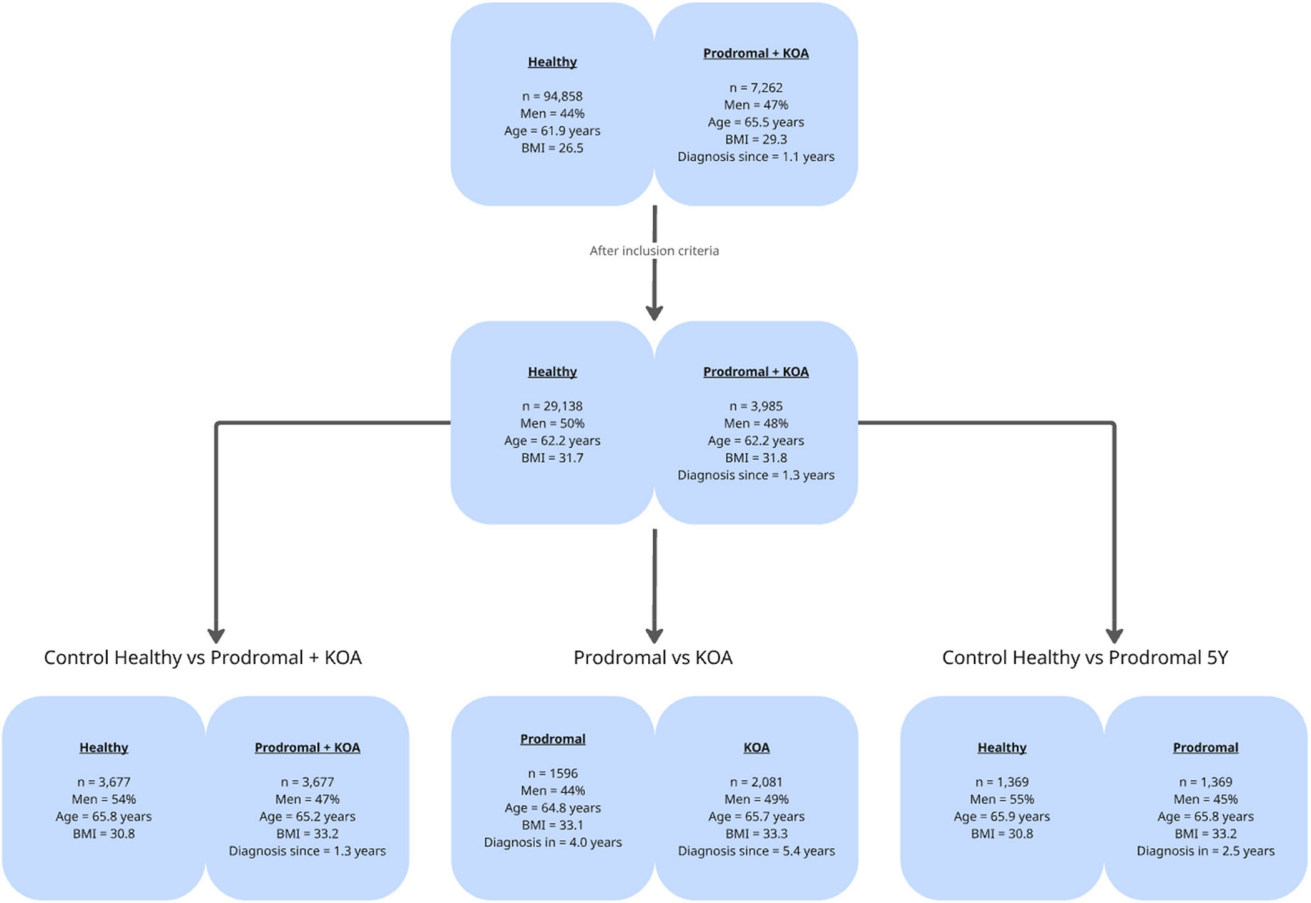
Participant selection flowchart and demographics. The diagram illustrates the data filtering process and population characteristics for the three classification scenarios analyzed. BMI, body mass index; KOA, knee osteoarthritis.

Among 102,120 participants with valid accelerometer data, we identified 7262 with KOA. We then performed three classification analyses as outlined above. After applying the inclusion criteria, 29,138 participants were classified as healthy and 3985 as KOA. Population demographics for each scenario are shown in Figure 4. For the Healthy versus Prodromal + KOA analysis, we included 3677 participants per group with a mean age of approximately 65.5 years. The Prodromal versus KOA comparison analyzed 1596 prodromal cases (mean age 64.8 years, 44% men) against 2081 diagnosed KOA cases (mean age 65.7 years, 49% men). Finally, the Control Healthy versus Prodromal 5Y subset matched 1369 healthy controls to 1369 prodromal cases diagnosed within 5 years, with both groups averaging 65.9 years of age. All predictive models achieved classification accuracies significantly above the random chance baseline of 50% (one‐sample *t* test, *n* = 5 folds, *p* < 0.001 for all comparisons), confirming that the identified accelerometer patterns contain predictive signal for KOA status beyond random noise.

### Healthy versus KOA

We compared accelerometer data from participants who never received a KOA diagnosis (Class 0) with participants who had a KOA diagnosis at any time or received one during follow‐up (Class 1). To create balanced cohorts, we randomly selected 3677 participants for each class. The groups were similar with respect to sex, age at recording and BMI (Figure 4).

**Figure 5:**
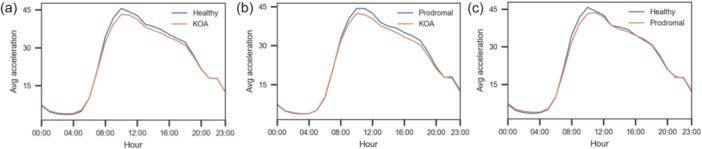
(a–c) Comparison of mean accelerometer values over a 24‐h cycle across different study groups. The plots illustrate diurnal activity patterns, displaying average acceleration on the *y*‐axis against the hour of the day (00:00–23:00) on the *x*‐axis. (a) Healthy controls (blue) versus KOA patients (orange). (b) Prodromal group (blue) versus KOA patients (orange). (c) Healthy controls (blue) versus Prodromal group (orange). All groups demonstrate a similar trend with peak activity occurring around 10:00, though distinct differences in movement intensity are observable between the classes throughout the active hours. KOA, knee osteoarthritis.

Figure 5a shows mean accelerometer values across 24 h for both classes. The y‐axis is average acceleration, and the *x*‐axis is hour of day (0–23). Both classes followed a similar diurnal pattern with peak activity around 10:00 and a gradual decline towards evening. Class 0 had slightly higher average acceleration than Class 1 throughout the day.

**Figure 6:**
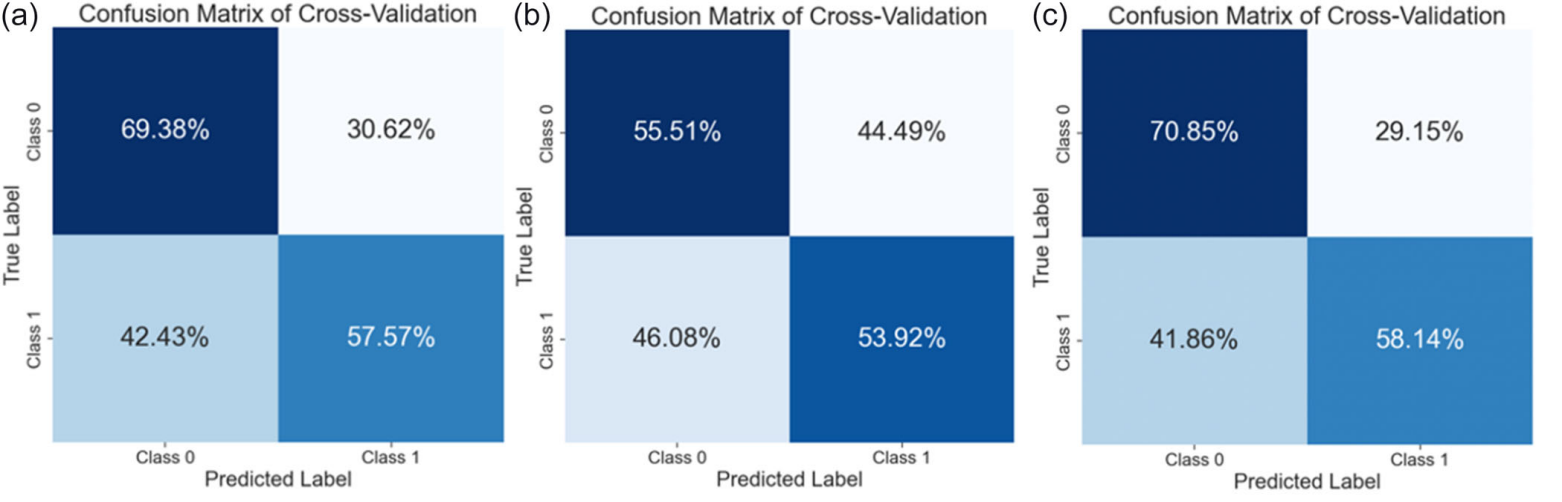
(a–c) Normalized confusion matrices for the best cross‐validation models. (a) KOA diagnosis (Class 0) versus KOA anytime/during follow‐up (Class 1). (b) Prodromal stage (Class 0) versus KOA diagnosis (Class 1). (c) Never diagnosed (Class 0) versus diagnosis within 5 years (Class 1). KOA, knee osteoarthritis.

Figure 6a shows the normalized confusion matrix of the best model. The model correctly classified 69% of Class 0 and 58% of Class 1. Misclassification occurred in 31% of Class 0 (predicted as Class 1) and 42% of Class 1 (predicted as Class 0).

Cross‐validation performance. Using fivefold cross‐validation (Table 1), the mean accuracy was 63.5%. The macro one‐vs‐rest AUC was 0.672. These results indicate stable, better‐than‐chance performance.

**Table 1:** Cross‐validation performance metrics for the three classification scenarios.

	Accuracy	F1‐score (macro)	Sensitivity (macro)	AUC (macro)	*p*
Healthy vs. KOA	63.5 ± 0.12%	0.633 ± 0.013	0.635 ± 0.012	0.672 ± 0.017	<0.0001
Prodromal vs. Diagnosed	54.6 ± 0.16%	0.543 ± 0.016	0.547 ± 0.017	0.552 ± 0.015	<0.0001
Healthy vs. Prodromal	64.5 ± 0.79%	0.643 ± 0.088	0.645 ± 0.079	0.682 ± 0.115	<0.0001

*Note*: Data are reported as mean ± standard deviation.

Abbreviations: AUC, area under the curve; KOA, knee osteoarthritis.

### Prodromal KOA versus diagnosed KOA

We compared participants in a prodromal stage of KOA (Class 0; *n* = 1596) with participants with a KOA diagnosis (Class 1; *n* = 2081). Prodromal status was defined as developing KOA within the observation window, specifically 0–10 years before clinical diagnosis. The groups were similar in sex, age at recording, and BMI (Figure 4).

Figure 5b shows mean accelerometer values over 24 h. Both classes had low values overnight (00:00–06:00), a sharp rise in the morning, and peak activity around 10:00, followed by a gradual decline. Class 0 showed slightly higher acceleration across the day.

Figure 6b shows the normalized confusion matrix of the best model. The model correctly classified 55.5% of Class 0 and 53.9% of Class 1. Misclassification occurred in 44.5% of Class 0 and 46% of Class 1. Performance was moderate with a slight advantage for Class 0.

Cross‐validation performance. With fivefold cross‐validation, mean accuracy was 54.6%. The macro one‐vs‐rest AUC was 0.552. Performance was consistent but modest across folds (Table 1).

### Healthy versus receiving KOA within 5 years

We compared participants who never received a KOA diagnosis (Class 0) with those who received a KOA diagnosis within 5 years of measurement (Class 1). We randomly selected 1369 participants per class. The groups were similar in sex, age at recording and BMI (Figure 4).

Figure 5c shows mean accelerometer values across the day. Both classes had low values overnight and early morning, a steep rise starting around 06:00, peak activity at 10:00–11:00, then a gradual decline. Class 0 showed slightly higher acceleration during active hours, especially 09:00–16:00.

Figure 6c shows the normalized confusion matrix of the best model. The model correctly identified 71% of Class 0 and 58% of Class 1. Misclassification occurred in 29% of Class 0 and 42% of Class 1.

Cross‐validation performance. With fivefold cross‐validation, the mean accuracy was 64.5%. The macro one‐vs‐rest AUC was 0.682. Performance was consistent across folds with moderate discriminative ability (Table 1).

## DISCUSSION

In our study, we hypothesize that daily wrist‐worn accelerometry contains movement patterns that differ between healthy people and KOA and may offer measurable signals associated with early KOA development. Our findings show that wrist‐worn accelerometry captures a detectable signal of KOA. Models differentiated healthy from current or prodromal KOA with moderate accuracy and predicted KOA within 5 years at a similar level, suggesting that this signal emerges well before diagnosis. By contrast, distinguishing prodromal from already diagnosed KOA was close to chance. This is consistent with the idea that prodromal individuals already exhibit KOA‐like movement patterns despite lacking diagnostic coding. To our knowledge, this is the first large study to use wrist accelerometry with ML for KOA prediction.

The study cohort (2014–2019) reflects national figures for height, obesity and sex distribution during that period. The 2018 Health Survey for England reported that 26% of men and 29% of women had a BMI over 30, with mean heights of 175.6 cm for men and 162.1 cm for women [16]. In our data, BMI strongly influenced overall activity. Because KOA cases had a mean BMI above 29 and excess body weight is a known risk factor for KOA development [12, 14, 18, 19], we restricted analyses to participants with a BMI ≥ 29 to limit confounding. For this reason, no reliable conclusions can be drawn from the results obtained here for subjects with a BMI < 29. Since accelerometer data were not available for the entire population of the UK Biobank data set, a possible bias, for example, regarding activity, cannot be ruled out.

Daily activity profiles were similar across groups, with familiar mid‐morning peaks and evening declines, yet healthy participants showed slightly higher average acceleration across the day. The model trained to separate healthy individuals from those with prevalent or incident KOA achieved a moderate discrimination (AUC 0.67). Specificity exceeded sensitivity, suggesting the model was better at recognizing those who remained KOA‐free than those who did or would develop KOA. This pattern likely reflects heterogeneous and subtle early changes in movement that are not fully captured at the wrist. The results support the idea that small shifts in free‐living activity may precede clinical diagnosis and could act as early digital markers, although accelerometry alone is not sufficient for clinical decision making.

We compared individuals who developed KOA within the next 0–10 years with those already diagnosed at recording, matched on age, sex, BMI, and height. In contrast to Group 1 (healthy vs. any KOA), differentiating prodromal from already diagnosed KOA showed no predictive value. Although prodromal participants displayed slightly higher activity levels, the model performed close to chance (AUC 0.55) and would even fall below a trivial baseline that would classify all individuals as ‘diagnosed’. KOA often progresses slowly over many years [19], so once KOA‐related movement changes are present, wrist‐level activity may look broadly similar whether a formal diagnosis has been recorded or not. These findings suggest that staging and timing relative to diagnosis are not well captured by wrist accelerometry alone and will likely require additional data types.

When restricting to individuals who either remained KOA‐free or received a diagnosis within 5 years, healthy participants again showed slightly higher daytime acceleration. The classifier achieved moderate discrimination with consistent cross‐validation results. Misclassification was more common among those who went on to develop KOA, which points to variable and subtle early signals. Prior work in Parkinson's disease has shown that one week of wrist accelerometry can outperform genetics, lifestyle, blood biomarkers and prodromal questionnaires for identifying both pre‐diagnostic and diagnosed cases [20]. Parkinson's disease produces marked upper‐limb motor signatures, while KOA primarily affects the lower limbs, which likely makes wrist‐based detection more challenging. Nielsen et al. developed an ML model predicting the 5‐year risk of osteoarthritis diagnosis, integrating retrospective clinical, lifestyle and biomarker data from the UK Biobank with ROC‐AUC of 0.73 for KOA [17]. Joint‐related models (like KOA) were only modestly different compared to the overall OA‐model (ROC‐AUC 0.72). BMI was ranked as the most important feature for the prediction of knee OA risk with ROC‐AUC of 0.65 of its own. Furthermore, model performance improved when additional biomarker features were included. It might well be that adding further biomarkers will also improve our model with an AUC of 0.68 accordingly. This should be taken into account for further studies.

In summary, three patterns emerge: First, models that contrast healthy participants with anyone who has or will have KOA reach moderate discrimination, indicating that much of the relevant signal is present in routine movement. Second, prediction of KOA within 5 years performs similarly, consistent with a signal that appears years before diagnostic coding. Third, distinguishing prodromal from already diagnosed KOA is close to chance, which fits a diagnostic‐lag hypothesis in which many prodromal participants already display KOA‐like activity but have not yet been diagnosed.

From a practical standpoint, even a single wrist device with unstructured accelerometer data, paired with ML and minimal clinical context, can provide population‐level risk stratification for KOA. KOA affects millions of people, and treatment pathways have changed little over time [18]. Early identification is therefore valuable. Compared with genetic testing or biomarker panels, accelerometers offer a low‐cost and widely available option, especially as many people already wear them in daily life.

This study used data from a single real‐world data set, where participants wore a wrist device during daily activities. While this improves real‐life applicability, the data was noisier. Including more data sets with diverse demographics could enhance model generalizability. Our models relied only on orientation‐independent acceleration magnitude, excluding axis‐specific data affected by device placement.

The current performance is not ready for individual clinical decisions but is useful for developing screening approaches and prioritizing further evaluation. Wrist accelerometry alone did not distinguish prodromal from diagnosed KOA, which remains the domain of clinical examination and knee‐focused imaging. Continued work that combines signals from multiple sensors, alternative body positions, and complementary clinical and biomarker data is likely to improve performance. But accelerometry remains a low‐cost and scalable option for population‐level triage, particularly because many people already wear such devices in everyday life.

Data were collected in free‐living conditions, which introduces noise, artefacts and missing values. Although preprocessing was applied, residual artefacts may remain, and filtering reduced the sample size. The data set did not include activity labels, which limited the interpretation of specific behaviours and required model‐based inference. Mapping disease status to signal patterns was therefore indirect. The UK Biobank cohort may also differ from the wider population, which limits generalizability.

We analyzed aggregated acceleration per hour over the week rather than the raw wrist accelerometer signal from the UK Biobank. In principle, analyzing raw data within walking segments would be preferable because it could capture gait speed, cadence, symmetry, and short bouts that may be relevant to knee pathology. This was not feasible here because the accelerometer data are not labelled, and current segmentation models for free‐living wrist data remain insufficiently accurate for reliable identification of walking in diverse daily contexts. Using hour‐level summaries reduced misclassification from noisy segment detection but also sacrificed temporal detail and likely attenuated signals tied to brief or task‐specific movements.

A wrist device may not capture the movements most relevant to knee pathology. Prior work suggests a single sensor at the thigh can be more informative for KOA [22]. Accelerometers measure linear acceleration only and do not capture rotational movement that can reflect limp or asymmetry. Adding gyroscopes, or using composite inertial measurement units, would likely provide richer information.

BMI and related factors influence activity patterns. Lee et al. reported that obesity, diet quality, severe pain, and severe dysfunction are associated with physical inactivity in adults with KOA [13]. In our data, clear activity differences between KOA and non‐KOA participants emerged from a BMI of 29 and upward. Below that threshold, BMI rather than KOA appeared to drive lower activity. We therefore focused on BMI ≥ 29 and cannot draw reliable conclusions for BMI < 29. Finally, our reliance on ICD‐10 hospital records to define KOA likely biases the ground truth towards severe cases seeking specialist care, potentially missing milder cases managed in primary care.

## CONCLUSION

Wrist‐worn accelerometry contains a reproducible signal associated with KOA, enabling moderate separation of healthy individuals from those with current or future KOA and identifying elevated risk up to 5 years before diagnosis. Prodromal and diagnosed KOA could not be distinguished, indicating that KOA‐related movement changes emerge long before clinical coding.

## AUTHOR CONTRIBUTIONS

All authors contributed to the study conception and design. Material preparation, data collection, analysis and final writing were performed by Ricardo Smits Serena. The first draft of the manuscript was written by Christina Valle, and all authors commented on previous versions of the manuscript. All authors read and approved the final manuscript.

## CONFLICT OF INTEREST STATEMENT

The authors declare no conflicts of interest.

## ETHICS STATEMENT

This research was performed on anonymized secondary data from the UK Biobank (Application ID 144927). All UK Biobank participants provided written informed consent for the use of their data in health‐related research, and the UK Biobank has obtained ethical approval from the relevant UK Research Ethics Committee. Because the analysis involved only existing, fully de‐identified data, no additional ethical approval was required in Germany for this secondary‐data analysis.

## Data Availability

The data that support the findings of this study are available from the UK Biobank under Application Number 144927. Researchers who wish to access the same data set must apply directly to the UK Biobank and comply with its Data Sharing Policy.
